# Large-Section Histopathology Can Better Indicate the Immune Microenvironment and Predict the Prognosis of Pancreatic Ductal Adenocarcinoma Than Small-Section Histopathology

**DOI:** 10.3389/fonc.2021.694933

**Published:** 2021-07-12

**Authors:** Guiling Ding, Meng Guo, Yelin Yang, Chen Sun, Shengyong Wu, Xingchen Liu, Jin Wang, Hui Jiang, Yanfang Liu, Jianming Zheng

**Affiliations:** ^1^ Department of Pathology, Shanghai General Hospital Affiliated to Shanghai Jiaotong University, Shanghai, China; ^2^ Department of Pathology, Changhai Hospital Affiliated to Navy Medical University (Second Military Medical University), Shanghai, China; ^3^ National Key Laboratory of Medical Immunology & Institute of Immunology, Naval Medical University (Second Military Medical University), Shanghai, China; ^4^ Department of Health Statistics, Naval Medical University (Second Military Medical University), Shanghai, China

**Keywords:** pancreatic ductal adenocarcinoma, tumor microenvironment, large histological sections, immune cell, prognosis, nomogram

## Abstract

Pancreatic ductal adenocarcinoma (PDAC) is a highly malignant tumor and is insensitive to radiotherapy and chemotherapy, as it is highly correlated with its complex tumor microenvironment (TME). A comprehensive description of PDAC’s immune microenvironment at the pathological level has not been reported, thus limiting its treatment. Previous studies have shown that large-section histopathology (LSH) can reveal the complete structure and margin of the tumor on a single slice and effectively reflect intratumoral heterogeneity. LSH, as opposed to classic small-section histopathology (SSH), can also be used to explore the infiltration state of immune cells in different regions. In the current study, EnVision immunohistochemical staining was used to explore the panoramic distribution of CD4-, CD8-, CD15-, CD20-, and CD56 (surface markers of helper T cells, cytotoxic T cells, neutrophils, B cells, and NK cells, respectively)-positive cells in 102 pairs of paraffin wax-embedded PDAC samples (LSH *vs* SSH) for the first time. These indicators were then analyzed, and correlations of clinicopathological characteristics with clinical prognoses were analyzed. The findings of this study show that LSH can effectively indicate more immune cells than SSH. Upregulated CD4, CD8, CD20, and CD56 or downregulated CD15 was correlated with a good prognosis in PDAC patients. However, analysis of SSH showed that only upregulated CD4 and CD8 can be used as indicators of a good prognosis. Multivariate Cox regression analysis showed that 7 variables, namely, pTNM stage (*P*=0.002), PDL1 expression (*P*=0.001), CDX2 expression (*P*=0.008), DPC4 expression (*P*=0.004), CD4 expression in LSH (*P*<0.001), CD8 expression in LSH (*P*=0.010) and CD15 expression in LSH (*P*=0.031), were significantly correlated with the prognosis of PDAC patients. The findings of this study indicate that LSH is an effective tool for a panoramic assessment of the immune microenvironment in pancreatic cancer patients.

## Introduction

Pancreatic ductal adenocarcinoma (PDAC) is associated with a high mortality rate and has a 5-year survival rate of less than 9% (1). Multidisciplinary surgical procedures (2), adjuvant and neoadjuvant therapies (3), and combined immunotherapy (4) are ineffective in the treatment of PDAC, partly because of its unique tumor microenvironment (TME). Pancreatic cancer contains several stromal cells with poor-quality angiogenesis and is accompanied by sustained hypoxia in the tumor, in contrast to other solid tumors. This special metabolic environment induces highly malignant tumor clones and causes nutrient depletion and metabolite accumulation in the microenvironment. The distribution and function of immune cells are affected by their interactions with other cellular components, resulting in an immunosuppressive microenvironment (5). Therefore, a panoramic view of immune cell composition in the TME is important for evaluating the prognosis of PDAC and for developing postoperative treatment strategies.

The composition of immune cells in the microenvironment of pancreatic cancer is complex and varies significantly among patients. Previous studies have shown that the infiltration of CD4^+^ T cells and CD8^+^ T cells in the microenvironment is associated with an excellent prognosis (6). In addition, Th1 cells among CD4^+^ T cells can activate CD8^+^ cytotoxic T lymphocytes (CTLs), thus stimulating the immune system to suppress tumor progression (5). Furthermore, NK cell infiltration indicates a good prognosis in PDAC patients (7). Tumors that escape T cell surveillance by downregulating MHC-I molecules after radiotherapy and chemotherapy can be killed by NK cells through the missing self recognition mechanism (8), which is an important process involved in tumor immunosurveillance. In addition, NK cells can kill tumor cells through an antibody-dependent cell-mediated cytotoxicity (ADCC) pathway by binding to tumor-related antibodies (8). Currently, the role of B cells in the microenvironment of pancreatic cancer is controversial. For example, a previous study reported that B cells can directly recognize tumor antigens and produce tumor-related antibodies that kill tumor cells by activating the complement system or by inducing ADCC effects (9). However, the sustained action of tumor-related antibodies can mediate the modulation of tumor antigens and facilitate tumor immune escape (10). The role of neutrophils in the microenvironment of pancreatic cancer is also controversial. Studies have shown that neutrophils inhibit tumor growth by releasing reactive oxygen species and reactive nitrogen species, promoting T cell activation, recruiting M1 macrophages, and activating ADCC effects (11). In addition, neutrophils can promote tumor growth by releasing matrix metalloproteinase 9, inhibiting NK cell activity, inducing CD8^+^ T cell apoptosis in the microenvironment, and recruiting Tregs (11). This current study focused on the role of CD4^+^ helper T cells, CD8^+^ cytotoxic T cells, CD15^+^ neutrophils, CD20^+^ B cells, and CD56^+^ NK cells in the TME.

Several techniques, such as the application of deconvolution algorithms to analyze RNA-seq data in tumor tissues (12), single-cell sequencing (13), spatial transcriptomics (14), and mass cytometry (15), have been used to explore immune cells in the TME. However, these techniques can be used on only local/target tumor samples, which may introduce biases owing to the different sampling sites of the pancreatic cancer. In the current study, large-section histopathology (LSH) refers to slices measuring 7.5 cm×5 cm×4 µm after processing large paraffin blocks utilizing a large format microtome, a large format cassette, and a large glass slide. The LSH technology prototype was initially used to study brain tumor pathology in the 1970s (16). However, the application of traditional LSH technology is limited by factors such as operation difficulties, a long production cycle, a complex process, high cost, and poor slice quality. New LSH techniques have been developed in recent years. These new techniques are effective in a variety of tumors, including breast cancer (17), prostate cancer (18), colorectal cancer (19), and oral squamous cell carcinoma (20). LSH can effectively present normal, diseased, and adjacent tissues, as well as multiple cutting edges on a slice. Therefore, LSH allows the better observation of lesions and a more objective and comprehensive evaluation than small-section histopathology (SSH). In addition, LSH findings can be compared with CT image findings, thus improving the overall understanding of this disease. In the present study, the use of the LSH technology system in pancreatic cancer was explored. CD4-, CD8-, CD15-, CD20-, and CD56-positive cell infiltration was detected in paired paraffin-embedded PDAC samples (LSH vs SSH) and was further correlated with clinical prognosis. Furthermore, a nomogram model was constructed to assess the survival rates of PDAC patients.

## Materials and Methods

### Preparation of Tissue Samples and Collection of Clinicopathological Data

A total of 102 specimens were obtained after radical resection of pancreatic cancer tissues at the General Surgery Department of Changhai Hospital Affiliated to the Second Military Medical University between August 2018 and January 2019. Patients included in this study did not receive any adjuvant treatment prior to surgery. All specimens were fixed with 10% neutral formalin, and biopsy samples were collected. Specimens were then divided into paired LSH (size 7.5 cm×5 cm×4 µm) and SSH (size 3.2 cm×2.4 cm×4 µm) samples. All patients, including 58 males and 44 females, aged between 28-82 years, with a median age of 63 years, were diagnosed with primary PDAC by senior pathologists. Data on 19 clinicopathological parameters, namely, gender, age, tumor location, tumor diameter, tumor differentiation degree, T, N and pTNM stage (TNM stage according to the 8^th^ edition of the American Joint Committee on Cancer (AJCC) staging system) (21), with or without peripancreatic lymph node metastases, number of peripancreatic lymph node metastases, with or without total lymph node metastases, number of total lymph node metastases, with or without neural invasion, with or without vascular tumor thrombus, with or without postoperative recurrence and metastases, and with or without postoperative chemoradiotherapy, and the levels of PDL1, CDX2 and DPC4 expression were recorded. Follow-up information was obtained using an electronic medical recording system and short messages. Follow-up started on the day after the operation and ended on October 31, 2020. Overall survival (OS) was defined as the time interval between the first day after the operation to death due to any cause or termination of follow-up. All participants signed an informed consent form prior to inclusion. This study was approved by the Ethics Committee of Changhai Hospital Affiliated to the Second Military Medical University.

### Immunohistochemistry (IHC) and Multiple Immunohistochemistry (mIHC) Analyses

All 102 specimens from PDAC patients were analyzed by IHC at the pathology department using the Envision method. PBS was used instead of the primary antibody as a negative control, and a known positive section was used as a positive control. Four-micrometer-thick whole-tissue wax sections were deparaffinized twice with xylene. Slices were then hydrated with 100, 95, and 85% ethanol and PBS (pH=7.3). Slices were incubated in preboiled EDTA (pH=8.0) at a high temperature and high pressure for antigen retrieval for 10 minutes. The heat source was immediately withdrawn followed by cooling to room temperature. Slices were placed in 3% H_2_O_2_ for 15 minutes to block endogenous peroxidase. The primary antibody was added to the sections in a wet box, followed by overnight incubation at 4°C. The secondary antibody was then added to the reaction, followed by 30 minutes of incubation at room temperature. Finally, tissues were counterstained with DAB, and nuclei were stained with hematoxylin. Sections were washed with PBS (pH=7.3) between all steps. The primary antibodies used in this study included those against CD4 (SP35), CD8 (SP16), CD15 (MMA), CD20 (L26), and CD56 (123C3.D5), all purchased from Fuzhou Maxin Biotechnologies Development Co., Ltd., and those against PDL1 (SP142), CDX2 (EP25), and DPC4 (B-8), all purchased from Beijing Zhongshan Jinqiao Biotechnology Co., Ltd. The DAB color kit (ZLI-9019) and secondary antibodies (goat anti-mouse/rabbit IgG polymer) were also purchased from Beijing Zhongshan Jinqiao Biotechnology Co., Ltd. (**Table 1**).

**Table 1:** Main reagents and instruments.

Reagent or resource	Source	Identification of product
IHC&mIHC: rabbit anti-CD4 (monoclonal, SP35)	MXB	RMA-0620
IHC&mIHC: rabbit anti-CD8 (monoclonal, SP16)	MXB	RMA-0514
IHC&mIHC: mouse anti-CD15 (monoclonal, MMA)	MXB	MAB-0779
IHC&mIHC: mouse anti-CD20 (monoclonal, L26)	MXB	Kit-0001
IHC&mIHC: mouse anti-CD56 (monoclonal, 123C3.D5)	MXB	Kit-0028
IHC: rabbit anti-PDL1 (monoclonal, SP142)	ZSGB-BIO	ZA-0629
IHC: rabbit anti-CDX2 (monoclonal, EP25)	ZSGB-BIO	ZA-0520
IHC: mouse anti-DPC4 (monoclonal, B-8)	ZSGB-BIO	ZM-0097
IHC: universal secondary antibody, goat anti-mouse/rabbit IgG polymer	ZSGB-BIO	PV-8000-1
IHC: DAB	ZSGB-BIO	ZLI-9019
IHC: EDTA (pH=8.0)	ZSGB-BIO	ZLI-9066
IHC: PBS (pH=7.3)	ZSGB-BIO	ZLI-9062
mIHC: AR9	Gene Tech	GTI00411
mIHC: Neon TSA 520	Yuanxibio	D110011
mIHC: Neon TSA 570	Yuanxibio	D110013
mIHC: Neon TSA 620	Yuanxibio	D110014
mIHC: Neon TSA 650	Yuanxibio	D110015
mIHC: Neon TSA 700	Yuanxibio	D110017
mIHC: DAPI	Thermo Fisher	D1306
mIHC: universal secondary antibody, goat anti-mouse/rabbit HRP polymer	Yuanxibio	A10011-60
Tissue imaging system	3DHISTECH	Pannoramic MIDI

mIHC staining was performed on 6 PDAC whole-tissue wax sections as follows. Four-micrometer-thick sections were deparaffinized, and antigen retrieval was performed as described for immunohistochemical analysis. Sections were incubated with standard primary antibodies, followed by the secondary antibody, using a TSA 6-color kit for mIHC. Sections were then counterstained with DAPI. Stained sections were dried at room temperature before image analysis. Sections were treated with 3% hydrogen peroxide solution to cover the sample area after deparaffinization and antigen retrieval. Sections were then incubated for 30 minutes with a mouse anti-CD20 antibody (L26, MXB). Furthermore, sections were incubated for 10 minutes with a goat anti-mouse/rabbit horseradish peroxidase (HRP) secondary antibody (#A10011-60, Yuanxibio). After incubation with antibodies, sections were flooded with Neon-TSA520 fluorescent solution and then incubated for 10 minutes at room temperature following the manufacturer’s instructions. Finally, sections were washed using TBST buffer and immediately transferred to preheated EDTA (90°C). Sections were heated at 20% power in a microwave oven for 15 minutes and then cooled to room temperature. Sections were thoroughly washed with Tris buffer between all steps. The process was repeated using the following antibodies/fluorescent dyes, in order: rabbit anti-CD4 (SP35, MXB)/TSA570, rabbit anti-CD8 (SP16, MXB)/TSA620, mouse anti-CD15 (MMA, MXB)/TSA650, and mouse anti CD56 (123C3. D5, MXB)/TSA700. DAPI (D1306, Thermo Fisher) was used to counterstain the cell nucleus. Sections were sealed on cover glass using transparent nail polish, dried at room temperature, and then photographed with a Pannoramic MIDI tissue imaging system (3DHISTECH). The results were analyzed using INDICA HALO software (**Table 1**).

### Interpretation of IHC and mIHC Results

Tumor regions (including neoplastic glands and neoplastic stroma) were used to evaluate LSH or SSH rather than normal tissue adjacent to the tumor. The evaluation criteria for each column of immune cells were as follows: upregulation of CD4, CD8, CD20, and CD56 in the cell membranes of infiltrating immune cells in the TME and upregulation of CD15 in the cytoplasm or cell membrane. To quantify the immune cell populations in LSH and SSH, a full scan of the immunohistochemically stained LSH and SSH samples was performed, and then a third-party senior pathologist who had no interest in this study first conducted a full-field double-blind observation of the infiltrated immune cells in LSH and SSH samples under a low-power microscope. During the process of reading the whole tissue section, the pathologist found different spatial distributions in five types of immune cells (tumor center, tumor margin, tumor stroma), and none were completely limited to one region (e.g., completely confined to the tumor margin). The pathologist also consulted and referenced relevant literature and then obtained images of 3-6 selected areas with abundant immune cell infiltrates under a high-power field (22). The images were then imported into ImageJ software for objective counting. The total and average numbers of the five types of immune cells were calculated separately under a high-power field (22). Cells were divided into high and low expression groups based on the median expression level. PDL1 is expressed in the membrane and cytoplasm of cancer cells. A value ≥5% was defined as positive (23). On the other hand, CDX2 is expressed in the nucleus of cancer cells. The expression of CDX2 was considered positive when the percentage of positive cells was >1% (24). DPC4 was rated as positive when more than 5% of the cells in the tumor tissue showed cytoplasmic/nuclear staining (25). HALO software was used to analyze the mIHC results and the number and percentage of positive cells in the whole section after scanning the whole slice on a 3DHISTECH scanner.

### Statistical Analysis

Statistical analyses were performed using IBM SPSS 22.0 and GraphPad Prism 8.0.2 software. A t-test was used to analyze normally distributed and homogeneous variables; otherwise, a nonparametric test was used. Chi-square or adjusted chi-square tests or Fisher’s exact probability tests were used to analyze classified variables. Measurement data were grouped by the median, whereas classified variables were grouped by category. The Kaplan-Meier method and log-rank test were used to analyze “time to endpoints”, whereas the Cox proportional hazards model was adopted for univariate and multivariate analyses. Finally, the “survival”, “rms”, “Hmisc”, “ggplot2”, and “timeROC” packages in R language version 4.0.4 were used to construct and verify a nomogram model of PDAC prognosis. *P* values <0.05 were considered statistically significant.

## Results

### Distribution and Expression of CD4, CD8, CD15, CD20, CD56, PDL1, CDX2, and DPC4 in PDAC

**Figure 1:**
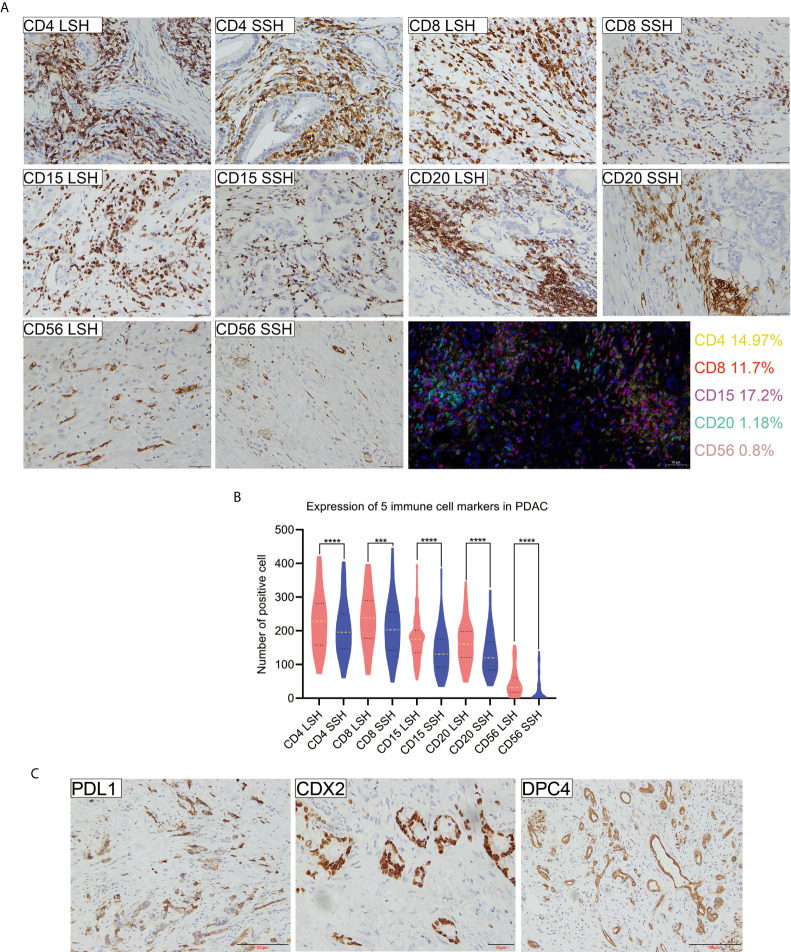
Expression profiles of 5 immune cell markers and PDL1, CDX2, and DPC4 in PDAC specimens. **(A)** Patterns of positive expression of CD4, CD8, CD15, CD20, and CD56 in LSH and SSH (scale bar=50 μm) and colocalization of 5 types of immune cells present in the TME of pancreatic head ductal adenocarcinoma in a 50-year-old man based on multiple immunohistochemical staining. Basic information of patients corresponding to CD4, CD8, CD15, CD20, and CD56 levels (LSH *vs* SSH): a 72-year-old man, a 61-year-old man, and a 66-year-old woman with a tumor located in the head of pancreas and a 54-year-old woman and a 67-year-old man with a tumor located in the tail of pancreas, respectively. **(B)** The expression levels of five immune cell markers were significantly higher in LSH than in SSH group (***P=0.0002, ****P<0.0001). **(C)** PDL1, CDX2, and DPC4 expression in PDAC. Basic information of patients corresponding to CDX2, DPC4, and PDL1 levels: a 50-year-old man with a tumor located in the head of the pancreas and a 66-year-old man and a 60-year-old man with a tumor located in the tail of the pancreas, respectively. CD4LSH and CD4SSH represent the levels of CD4 expression in LSH and SSH, respectively, and other abbreviations can be deduced by analogy. PDAC, pancreatic ductal adenocarcinoma.

To assess whether there were any differences in the detection of CD4, CD8, CD15, CD20, and CD56 by LSH and SSH, we analyzed and compared the expression levels of these markers across the respective panoramic images (**Supplementary Figures 1** and **2**) after IHC staining. Both LSH and SSH successfully revealed cells expressing CD4, CD8, CD15, CD20, and CD56 (**Figure 1A**). LSH (**Supplementary Figure 3**) better represented the panoramic information of the original tumor and completely present normal, diseased, and adjacent tissues, as well as multiple related cutting edges on a slice. Consequently, compared with SSH (**Supplementary Figure 3**), LSH not only conferred better visualization of lesions and a more objective and comprehensive evaluation but also provided more abundant spatial location information on immune cells. However, there was little difference in the distribution of immune cells between the two groups observed under the microscope. In LSH and SSH, CD4^+^ T cells often gathered around neoplastic glands or between neoplastic glands and stroma, CD8^+^ T cells were often densely clustered in the stroma far away from neoplastic glands or scattered around neoplastic glands to a small extent, CD15^+^ neutrophils were mostly scattered in the stroma or partially gathered around neoplastic glands, CD20^+^ B cells were often distributed in clusters around the neoplastic glands or scattered in the stroma, and CD56^+^ NK cells were often sparsely distributed in the stroma or near the neoplastic glands very few. A paired-sample Wilcoxon signed-rank test revealed significantly higher expression of the five immune cell markers in the LSH group than in the SSH group (*P*<0.001) (**Figure 1B**). Multiple immunohistochemical staining of PDAC whole tissue sections revealed coexpression of the 5 immune cell types in the TME, although their concentrations were differentially distributed across regions. In addition, these immune cells were not colocalized in the same area. Among them, CD4 (14.97%), CD8 (11.7%), and CD15 (17.2%) had higher proportions than CD20 (1.18%) and CD56 (0.8%) (**Figure 1A**). The rates of PDL1, CDX2, and DPC4 expression in whole tissue sections of the 102 PDAC specimens were 5.9, 11.8, and 6.9%, respectively (**Figure 1C**).

### Expression of Immune Markers Is Correlated With Clinicopathological Characteristics

We used a chi-square test to explore differences between the expression of the five immune cell markers and the clinicopathological features of PDAC in 102 patients based on LSH and SSH. Based on LSH, CD4 expression was significantly associated with tumor location (*P=*0.045), tumor differentiation (*P=*0.007), vascular tumor thrombus (*P=*0.008), postoperative chemoradiotherapy (*P*=0.017), and CDX2 expression (*P=*0.011) in PDAC patients. On the other hand, CD8 expression exhibited a significant correlation with postoperative chemoradiotherapy (*P=*0.013) and PDL1 expression (*P=*0.035), whereas CD15 expression was significantly associated with the pTNM stage of PDAC (*P=*0.029). Moreover, CD20 expression was significantly correlated with tumor location (*P=*0.007), tumor differentiation (*P=*0.005), and CDX2 expression (*P=*0.014) in PDAC patients. Conversely, we found no significant correlation between CD56 expression and any of the clinicopathological characteristics of PDAC (**Table 2**).

**Table 2:** Relationship between CD4, CD8, CD15, CD20, CD56, PDL1, CDX2, and DPC4 expression and clinicopathologic features of PDAC.

Characteristic	N=102	CD4 LSH expression		*P*	CD4 SSH expression		*P*	CD8 LSH expression		*P*	CD8 SSH expression		*P*	CD15 LSH expression		*P*	CD15 SSH expression		*P*	CD20 LSH expression		*P*	CD20 SSH expression		*P*	CD56 LSH expression		*P*	CD56 SSH expression		*P*
		Low	High		Low	High		Low	High		Low	High		Low	High		Low	High		Low	High		Low	High		Low	High		Low	High	
Gender																															
Male	58	28	30	0.530	28	30	0.530	27	31	0.424	29	29	1.000	28	30	0.689	31	27	0.424	31	27	0.424	32	26	0.331	36	22	0.258	45	13	0.122
Female	44	24	20		24	20		24	20		22	22		23	21		20	24		20	24		20	24		32	12		28	16	
Age (years)																															
≤63	50	24	26	0.555	25	25	0.846	22	28	0.235	27	23	0.428	27	23	0.428	25	25	1.000	24	26	0.692	22	28	0.167	35	15	0.484	36	14	0.925
>63	52	28	24		27	25		29	23		24	28		24	28		26	26		27	25		30	22		33	19		37	15	
Tumor location																															
Head/neck	65	38	27	0.045*	32	33	0.639	33	32	0.837	31	34	0.537	30	35	0.303	28	37	0.064	39	26	0.007*	38	27	0.045*	47	18	0.109	45	20	0.488
Body/tail	37	14	23		20	17		18	19		20	17		21	16		23	14		12	25		14	23		21	16		28	9	
Tumor diameter (cm)																															
≤3.5	65	33	32	0.955	33	32	0.955	33	32	0.837	34	31	0.537	34	31	0.537	31	34	0.537	36	29	0.149	34	31	0.722	44	21	0.771	47	18	0.826
>3.5	37	19	18		19	18		18	19		17	20		17	20		20	17		15	22		18	19		24	13		26	11	
Tumor differentiation																															
Well	83	37	46	0.007*	36	47	0.001*	39	44	0.204	38	45	0.075	41	42	0.799	43	40	0.445	36	47	0.005*	41	42	0.504	54	29	0.472	59	24	0.821
Poor	19	15	4		16	3		12	7		13	6		10	9		8	11		15	4		11	8		14	5		14	5	
pTNM stage																															
I+II	16	6	10	0.240	6	10	0.240	5	11	0.102	6	10	0.276	12	4	0.029*	8	8	1.000	8	8	1.000	8	8	0.932	10	6	0.700	11	5	1.000
III+IV	86	46	40		46	40		46	40		45	41		39	47		43	43		43	43		44	42		58	28		62	24	
T stage																															
T1-T2	77	40	37	0.732	39	38	0.907	37	40	0.490	38	39	0.818	42	35	0.107	40	37	0.490	37	40	0.490	36	41	0.134	53	24	0.416	56	21	0.649
T3-T4	25	12	13		13	12		14	11		13	12		9	16		11	14		14	11		16	9		15	10		17	8	
N stage																															
N0-1	50	22	28	0.167	22	28	0.167	24	26	0.692	26	24	0.692	29	21	0.113	30	20	0.048*	23	27	0.428	27	23	0.550	31	19	0.327	33	17	0.221
N2	52	30	22		30	22		27	25		25	27		22	30		21	31		28	24		25	27		37	15		40	12	
Peripancreatic lymph node metastases																															
No	24	11	13	0.564	11	13	0.564	11	13	0.641	13	11	0.641	16	8	0.062	14	10	0.350	12	12	1.000	13	11	0.721	13	11	0.137	12	12	0.007*
Yes	78	41	37		41	37		40	38		38	40		35	43		37	41		39	39		39	39		55	23		61	17	
Number of peripancreatic lymph node metastases																															
≤3	56	25	31	0.158	24	32	0.070	28	28	1.000	28	28	1.000	31	25	0.233	31	25	0.233	27	29	0.691	30	26	0.564	34	22	0.159	37	19	0.174
?3	46	27	19		28	18		23	23		23	23		20	26		20	26		24	22		22	24		34	12		36	10	
Total lymph node metastasis																															
No	22	10	12	0.558	10	12	0.558	10	12	0.630	12	10	0.630	15	7	0.054	14	8	0.149	11	11	1.000	12	10	0.706	13	9	0.395	12	10	0.046*
Yes	80	42	38		42	38		41	39		39	41		36	44		37	43		40	40		40	40		55	25		61	19	
Total number of lymph node metastases																															
≤3.5	51	22	29	0.113	22	29	0.113	24	27	0.552	26	25	0.843	30	21	0.075	31	20	0.029*	24	27	0.552	28	23	0.428	31	20	0.208	34	17	0.272
>3.5	51	30	21		30	21		27	24		25	26		21	30		20	31		27	24		24	27		37	14		39	12	
Neural invasion																															
No	4	1	3	0.582	3	1	0.638	1	3	0.610	2	2	1.000	3	1	0.610	3	1	0.610	1	3	0.610	2	2	1.000	4	0	0.367	4	0	0.471
Yes	98	51	47		49	49		50	48		49	49		48	50		48	50		50	48		50	48		64	34		69	29	
Vascular tumor thrombus																															
No	60	24	36	0.008*	28	32	0.298	27	33	0.227	31	29	0.687	31	29	0.687	35	25	0.044*	26	34	0.108	30	30	0.813	40	20	1.000	41	19	0.387
Yes	42	28	14		24	18		24	18		20	22		20	22		16	26		25	17		22	20		28	14		32	10	
Postoperative recurrence and metastases																															
No	26	12	14	0.568	12	14	0.568	11	15	0.363	9	17	0.069	16	10	0.173	11	15	0.363	12	14	0.650	12	14	0.568	17	9	0.872	16	10	0.189
Yes	76	40	36		40	36		40	36		42	34		35	41		40	36		39	37		40	36		51	25		57	19	
Postoperative chemoradiotherapy																															
No	20	15	5	0.017*	9	11	0.551	15	5	0.013*	10	10	1.000	10	10	1.000	10	10	1.000	8	12	0.318	9	11	0.551	16	4	0.158	16	4	0.351
Yes	82	37	45		43	39		36	46		41	41		41	41		41	41		43	39		43	39		52	30		57	25	
PDL1																															
Negative	96	47	49	0.225	48	48	0.710	47	51	0.035*	47	49	0.674	49	47	0.674	49	47	0.674	47	49	0.674	49	47	1.000	63	33	0.655	67	29	0.261
Positive	6	5	1		4	2		6	0		4	2		2	4		2	4		4	2		3	3		5	1		6	0	
CDX2																															
Negative	90	50	40	0.011*	47	43	0.492	48	42	0.065	47	43	0.219	43	47	0.219	44	46	0.539	49	41	0.014*	49	41	0.055	62	28	0.328	66	24	0.458
Positive	12	2	10		5	7		3	9		4	8		8	4		7	5		2	10		3	9		6	6		7	5	
DPC4																															
Negative	95	49	46	0.957	50	45	0.402	47	48	1.000	47	48	1.000	45	50	0.117	48	47	1.000	47	48	1.000	48	47	1.000	64	31	0.890	68	27	1.000
Positive	7	3	4		2	5		4	3		4	3		6	1		3	4		4	3		4	3		4	3		5	2	

*P < 0.05

Based on SSH, CD4 expression was significantly correlated with tumor differentiation *(P=*0.001), whereas CD15 was significantly associated with N stage (*P=*0.048), the number of PDAC patients with total lymph node metastases (*P=*0.029) and vascular tumor thrombus (*P=*0.044). On the other hand, CD20 expression was significantly correlated with tumor location (*P=*0.045) in PDAC patients, and CD56 expression was significantly correlated with peripancreatic (*P=*0.007) and total (*P=*0.046) lymph node metastases. Conversely, CD8 expression was not significantly correlated with any of the clinicopathological characteristics of PDAC (**Table 2**). In summary, except for CD56 in LSH and CD8 in SSH, the other four immune cell markers were all significantly associated with the clinicopathological characteristics of PDAC, but their degrees of correlations differed. These results suggest that postoperative samples from the same patient may lead to different research results due to different tissue sampling methods used (LSH and SSH). LSH may contain more organizational information.

### Immune Markers Are Correlated With Clinicopathological Characteristics and Prognosis

Univariate and multivariate Cox analyses were used to explore the relationship between 19 clinicopathological parameters and 10 immune cell parameters and the prognosis of PDAC patients. The following immune markers were examined to determine whether LSH or SSH is more representative of the original tumor and which better indicates patient prognosis: CD4 in LSH (CD4LSH), CD4 in SSH (CD4SSH), CD8 in LSH (CD8LSH), CD8 in SSH (CD8SSH), CD15 in LSH (CD15LSH), CD15 in SSH (CD15SSH), CD20 in LSH (CD20LSH), CD20 in SSH (CD20SSH), CD56 in LSH (CD56LSH), and CD56 in SSH (CD56SSH).

**Figure 2:**
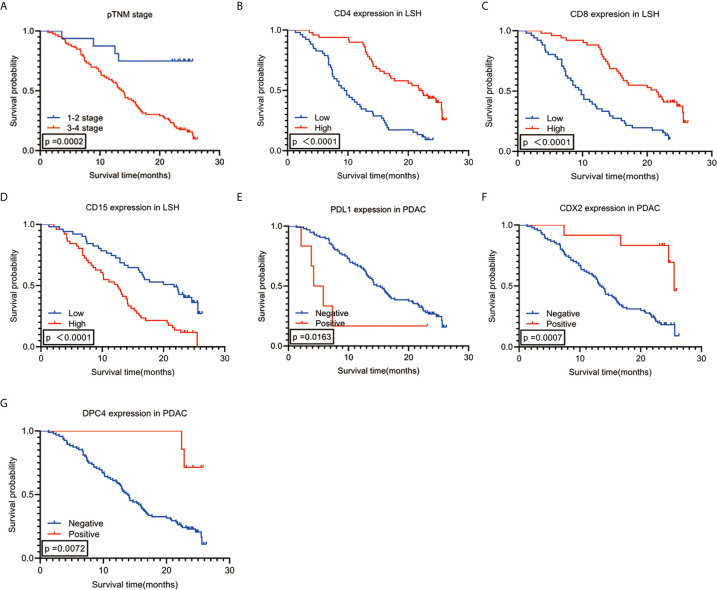
Kaplan-Meier survival analysis curves according to 7 **(A–G)** variables that were significantly associated with PDAC prognosis. PDAC, pancreatic ductal adenocarcinoma.

The enter screening method in the univariate Cox analysis showed a significant correlation between 21 factors and prognostic predictors: age (*P*=0.030), tumor differentiation (*P*<0.001), pTNM stage (*P=*0.001), N stage (*P=*0.001), with or without peripancreatic lymph node metastases (*P=*0.010), number of peripancreatic lymph node metastases (*P=*0.001), with or without total lymph node metastases (*P*=0.017), number of total lymph node metastases (*P*=0.001), vascular tumor thrombus (*P*=0.005), with or without postoperative recurrence and metastases (*P*=0.003), with or without postoperative chemoradiotherapy (*P*=0.003), PDL1 (*P*=0.022), CDX2 (*P*=0.002), DPC4 (*P*=0.017), CD4LSH (*P*<0.001), CD4SSH (*P*=0.039), CD8LSH (*P*<0.001), CD8SSH (*P*=0.013), CD15LSH (*P*<0.001), CD20LSH (*P*<0.001), and CD56LSH (*P*=0.043) (**Table 3**). The forward LR screening method in the multivariate Cox analysis was used to explore the 21 relevant parameters, and 7 independent factors associated with prognosis were identified (**Table 3**): pTNM stage (*P*=0.002), CD4LSH (*P*<0.001), CD8LSH (*P*=0.010), CD15LSH (*P*=0.031), PDL1 (*P*=0.001), CDX2 (*P*=0.008), and DPC4 (*P*=0.004). The 7 independent factors did not include any immune cell parameters in SSH. These findings imply that LSH effectively reflects the original tumor status and can effectively predict the prognosis of PDAC patients. Moreover, the Kaplan-Meier method and log-rank test were used to generate survival curves of OS-related parameters (**Figure 2** and **Supplementary Figure 4**).

**Table 3:** Univariate and multivariate analyses of the clinicopathological parameters and prognosis of PDAC patients in this study cohort.

Parameter	Univariate analysis			Multivariate analysis		
	*HR*	95% CI	*P*	*HR*	95% CI	*P*
Gender (Male *vs* Female)	0.762	0.483-1.200	0.240			
Age (≤63 years *vs* >63 years)	1.646	1.050-2.582	0.030*			
Tumor location (Head/neck *vs* Body/tail)	0.791	0.495-1.263	0.327			
Tumor diameter (≤3.5 cm *vs* >3.5 cm)	1.025	0.643-1.633	0.918			
Tumor differentiation (Well *vs* Poor)	3.105	1.811-5.321	<0.001*			
pTNM stage (I-II *vs* III-IV)	5.442	1.982-14.941	0.001*	5.713	1.939-16.832	0.002*
T stage (T1-T2 *vs* T3-T4)	1.049	0.617-1.783	0.859			
N stage (N0-N1 *vs* N2)	2.198	1.385-3.487	0.001*			
Peripancreatic lymph node metastasis (No *vs* Yes)	2.209	1.208-4.040	0.010*			
Number of peripancreatic lymph node metastases (≤3 *vs* >3)	2.132	1.352-3.361	0.001*			
Total lymph node metastasis (No *vs* Yes)	2.133	1.145-3.972	0.017*			
Total number of lymph node metastases (≤3.5 *vs* >3.5)	2.233	1.409-3.537	0.001*			
Neural invasion (No *vs* Yes)	5.301	0.736-38.194	0.098			
Vascular tumor thrombus (No *vs* Yes)	1.925	1.223-3.029	0.005*			
Postoperative recurrence and metastases (No *vs* Yes)	2.679	1.409-5.093	0.003*			
Postoperative chemoradiotherapy (No *vs* Yes)	0.453	0.269-0.763	0.003*			
CD4 expression in LSH (Low *vs* High)	0.297	0.184-0.479	<0.001*	0.360	0.211-0.612	<0.001*
CD4 expression in SSH (Low *vs* High)	0.618	0.391-0.976	0.039*			
CD8 expression in LSH (Low *vs* High)	0.352	0.221-0.563	<0.001*	0.511	0.306-0.853	0.010*
CD8 expression in SSH (Low *vs* High)	0.554	0.348-0.881	0.013*			
CD15 expression in LSH (Low *vs* High)	2.459	1.544-3.914	<0.001*	1.707	1.050-2.776	0.031*
CD15 expression in SSH (Low *vs* High)	1.261	0.808-1.967	0.308			
CD20 expression in LSH (Low *vs* High)	0.414	0.260-0.661	<0.001*			
CD20 expression in SSH (Low *vs* High)	0.745	0.476-1.165	0.197			
CD56 expression in LSH (Low *vs* High)	0.603	0.369-0.985	0.043*			
CD56 expression in SSH (Low *vs* High)	0.806	0.477-1.361	0.419			
PDL1 (Negative *vs* Positive)	2.908	1.165-7.257	0.022*	5.455	1.934-15.388	0.001*
DPC4 (Negative *vs* Positive)	0.180	0.044-0.737	0.017*	0.114	0.026-0.498	0.004*
CDX2 (Negative *vs* Positive)	0.203	0.073-0.559	0.002*	0.247	0.087-0.699	0.008*

*P < 0.05.

### Independent Prognostic Parameters of PDAC Are Associated With OS

**Figure 3:**
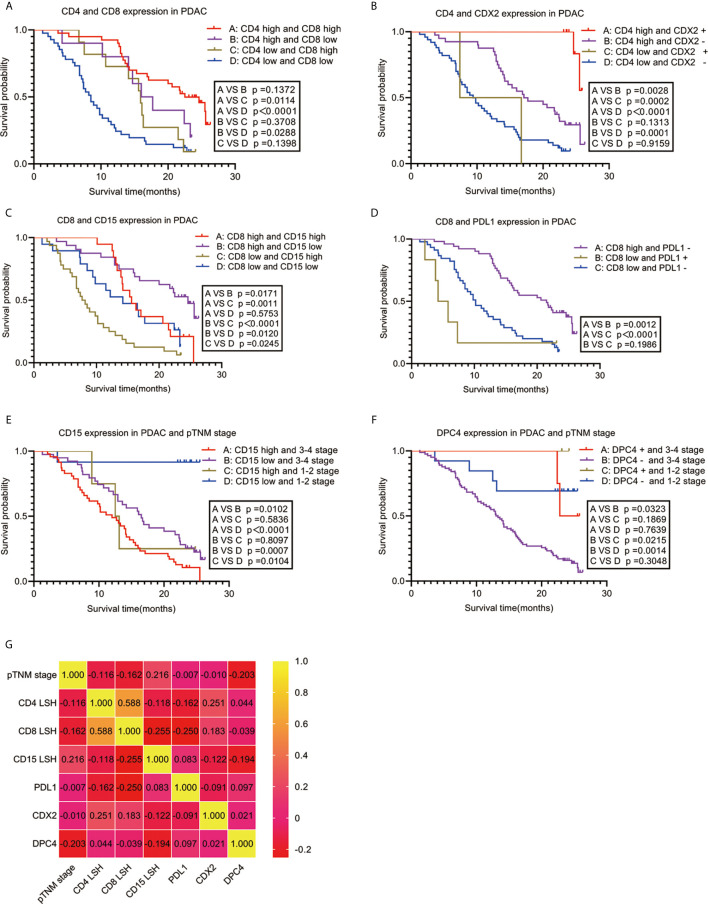
Correlation between PDAC-independent prognostic parameters and their combinations with OS. Survival analysis **(A–F)** and a heat map of the correlation coefficient matrix **(G)** of correlated combination variables for the prediction of PDAC prognosis. PDAC, pancreatic ductal adenocarcinoma.

Spearman correlation was used to analyze the relationship between the seven independent prognostic predictors of PDAC and OS. The findings showed a significant positive correlation between OS and high expression levels of CD4 and CD8 (r=0.588, *P*<0.001) and CDX2 positivity (r=0.251, *P*=0.011). Notably, a high expression level of CD8 was negatively correlated with high CD15 expression (r=-0.255, *P*=0.010) and PDL1 expression (r=-0.250, *P*=0.011). Analysis of the clinical stage showed that an early pTNM stage was correlated with low CD15 expression (r=0.216, *P*=0.029), whereas an advanced pTNM stage was correlated with DPC4 negativity (r=-0.203, *P*=0.041) (**Figure 3G**). Survival analysis using the Kaplan-Meier method and log-rank test showed a significant correlation among several combinations of variables (**Figures 3A–F**). Notably, analysis of the combination of CD4 and CD8 showed that the group with the best prognosis had high expression of CD4 and CD8, with a median OS period of 22.500 months (95% CI: 18.767-26.233, *P*<0.001), whereas the group with the worst prognosis had low expression of CD4 and CD8, with a median OS period of 8.500 months (95% CI: 6.623-10.377, *P*<0.001). Analysis of the combination of CD4 and CDX2 showed that the group with the best prognosis had high CD4 expression and CDX2 positivity. However, there was no median OS because only 2 of 10 patients in this group had an endpoint event at the end of follow-up. The average OS period for the group with the CD4 and CDX2 combination was 25.572 months (95% CI: 25.182-25.963, *P*<0.001). On the other hand, the group with the worst prognosis had low CD4 expression and CDX2 negativity. This group had a median OS period of 9.400 months (95% CI: 7.420-11.380, *P*<0.001). When CD8 was combined with CD15, the group with the best prognosis was the one with high CD8 expression and low CD15 expression, with a median OS period of 24.600 months (95% CI: 21.617-27.583, *P*<0.001). Conversely, the group with the worst prognosis exhibited low CD8 expression and high CD15 expression, with a median OS period of 7.700 months (95% CI: 5.760-9.640, *P*<0.001). When CD8 was combined with PDL1, the group with the best prognosis had high CD8 expression and PDL1 negativity. This group had a median OS period of 21.500 months (95% CI: 16.137-26.863, *P*<0.001). On the other hand, low CD8 expression and PDL1 positivity were associated with the worst prognosis, with a median OS period of 4.200 months (95% CI: 1.800-6.600, *P*<0.001). When CD15 was combined with pTNM stage, the group with the best prognosis had low CD15 expression and pTNM stages I-II. This group had no median OS, as only 1 of 12 people in this group had an endpoint event at the end of follow-up. The average OS period for this group was 23.675 months (95% CI: 20.250-27.100, *P*<0.001). Conversely, the group with the worst prognosis associated with this combination had high CD15 expression and pTNM stages III-IV, with a median OS period of 12.000 months (95% CI: 8.753-15.247, *P*<0.001). When DPC4 was combined with pTNM stage, the group with the best prognosis had DPC4 positivity and pTNM stages I-II. Notably, the median and average OS periods were not calculated due to a lack of terminal events among the 3 patients in this group at the end of follow-up (*P*<0.001). On the other hand, the group with the worst prognosis had DPC4 negativity and pTNM stages III-IV. This group had a median OS period of 13.000 months (95% CI: 11.136-14.864, *P*<0.001).

### Nomogram Model Construction and Verification

**Figure 4:**
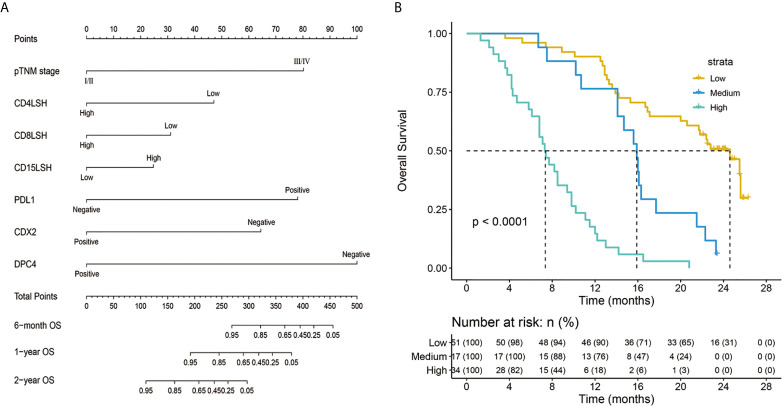
Nomogram predicting the survival rates of PDAC patients at 6 months, 1 year, and 2 years after surgery **(A)** and a survival curve of individual risk stratification based on the nomogram **(B)**. pTNM-pathology tumor-node-metastasis; CD4LSH, CD4 expression in large-section histopathology; CD8LSH - CD8 expression in large-section histopathology; CD15LSH, CD15 expression in large-section histopathology; PDL1, programmed cell death-ligand 1; CDX2, caudal-type homeobox 2; DPC4, deleted in pancreatic carcinoma 4.

**Figure 5:**
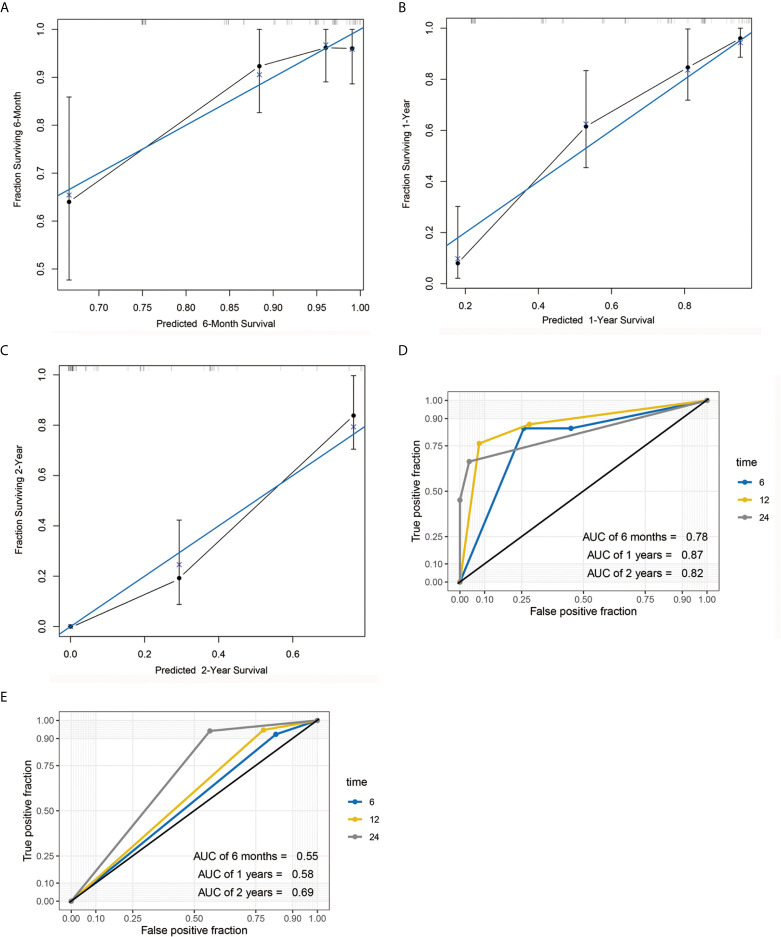
Calibration curve and AUC graph. Calibration curve for predicting the survival rates of PDAC patients at 6 months **(A)**, 1 year **(B)** and 2 years **(C)** after surgery. Blue and black lines represent the ideal calibration curve model and the actual calibration curve, respectively. AUC graph of the nomogram **(D)** and TNM staging system **(E)** for the predicting postoperative 6-month, 1-year, and 2-year survival rates.

**Figure 6:**
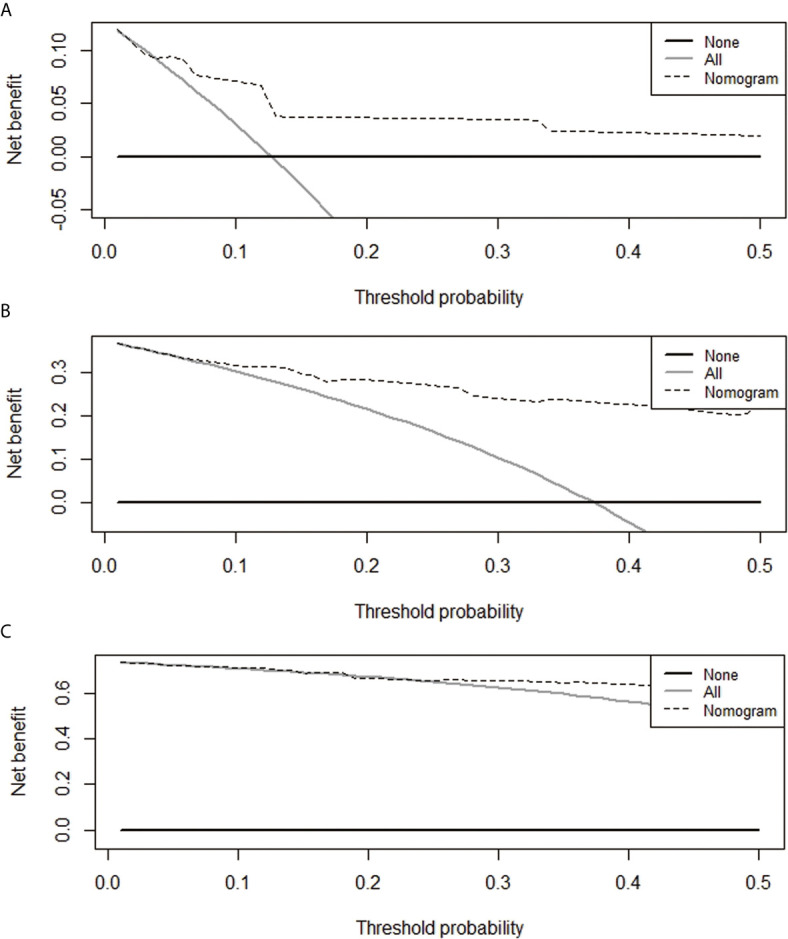
Decision curves for the prediction of postoperative survival rates of PDAC patients at 6 months **(A)**, 1 year **(B)**, and 2 years **(C)**.

A nomogram model was constructed based on the 7 variables obtained from the multivariate Cox analysis (pTNM stage, CD4LSH, CD8LSH, CD15LSH, PDL1, CDX2, and DPC4). The nomogram accurately predicted the prognosis of PDAC patients (**Figure 4A**). The risk scoring system of the nomogram classified patients into three groups, namely, low- (≤275), medium- (275–320), and high-risk (>320). Survival analysis using Kaplan-Meier curves and the log-rank test showed significant differences among the three groups (*P*<0.001) (**Figure 4B**). Bootstrap self-sampling (1000 times) was used to internally verify the nomogram. The C-index value was 0.80 (95% CI: 0.73-0.85), indicating that the model has high prediction accuracy. The area under the curve (AUC) values of the model were 0.78, 0.87, and 0.82 in PDAC patients at 6 months, 1 year, and 2 years after surgery, respectively (**Figure 5D**). The predicted AUC values for TNM stage were 0.55, 0.58, and 0.69 at 6 months, 1 year and 2 years after surgery, respectively (**Figure 5E**). These findings indicate that the nomogram model has a good degree of discrimination. Moreover, the AUC values predicted by the nomogram were all higher than those obtained using the TNM staging system. Calibration curves and baseline scores were similar, indicating that the model-predicted observations were consistent with actual observations (**Figures 5A–C**). In addition, decision curve analysis showed that the nomogram has good clinical value within a reasonable threshold probability range (**Figures 6A–C**).

## Discussion

Immune cells in the TME play a key role in the development and progression of pancreatic cancer (26). Pancreatic cancer, similar to other solid tumors, evades host immune surveillance by modulating immune cells to establish an immunosuppressive TME (27). T cells are the most representative type of immune cell in the TME and play an important role in the clinical outcome of pancreatic cancer (6, 28). Previous studies have shown that limited T cell infiltration occurs in primary PDAC mouse models, whereas insignificant levels of CD8^+^ T cells are present in tumor cell nests or the surrounding stroma (29). PDAC is an immunologically cold tumor characterized by sparse T cell infiltrates (30). PDAC patients with an abundance of T cells in the stroma and higher levels of CD4^+^ T and/or CD8^+^ T cells exhibit significantly longer survival times than those with lower levels of these immune cells (31). Previous studies have reported inconsistent findings, possibly due to bias from tumor tissue sampling or the analysis/technique used. Advances in detection technology have significantly improved research on the TME. For instance, the use of high-throughput sequencing technologies, such as deconvolution RNA-Seq and single-cell sequencing, has generated important omics resources. However, the long cycle times, high costs involved, and loss of spatial information during the analysis process negatively affect the effectiveness of these techniques. Spatial transcriptomics enables simultaneous labeling and high-resolution microscopic imaging of multiple antibodies on tissue slices, data analysis and mining experimental images after staining and is a recent technique used in studies on the TME. However, this technique is limited by its insufficient resolution; therefore, it does not meet the sequencing depth requirement and is associated with a high cost of single-cell sequencing. LSH offers the best solution to circumvent these limitations. The findings of the current study are consistent with findings from previous studies showing that T cells are one of the most representative types of immune cells in the pancreatic cancer TME. In addition, the findings of the current study showed that patients with higher levels of CD4^+^ T and/or CD8^+^ T cells have significantly longer survival times, consistent with previous findings (6, 31). A comparison between LSH and SSH in the detection of CD4^+^ T and CD8^+^ T cells in paired PDAC samples showed abundant CD4^+^ T and CD8^+^ T cell infiltration in LSH. Moreover, the findings using this technique showed independent factors that were significantly correlated with the prognosis of PDAC patients. This may be related to the Th1 cells among CD4^+^ T cells that activate CD8^+^ CTLs and promote the immune system to suppress tumor progression (5). A previous study explored the spatial distribution and functional status of T cells in different areas, including the tumor center, invasive front, normal parenchyma adjacent to the tumor, and tumor-positive and tumor-negative draining lymph nodes in PDAC (32). However, the current study explored only the infiltration of T cells in the PDAC tumor region and did not explore the functional status of T cells. Therefore, further studies should analyze the functional status of T cells. However, the present study showed that high levels of infiltrating T cells and low expression of PDL1 in PDAC were associated with a poor prognosis. These findings indicate that an abundance of T cell infiltrates in PDAC is an effective predictor of patient prognosis.

NK cells represent an important part of tumor immune monitoring. However, their occurrence in pancreatic cancer and their correlation with the prognosis of PDAC are still unclear. Previous studies explored few NK cells in partial pancreatic cancer samples and reported that pancreatic tumor cells have selective resistance to NK cell-mediated immune surveillance (7). Other studies reported high levels of NK cells in pancreatic cancer specimens based on tissue chip technology. The findings showed that a high concentration of CD56^+^ NK cells significantly correlated with a good prognosis in patients who had not received adjuvant chemotherapy (33). Notably, these findings are not consistent, possibly due to differences in tumor tissue sampling. The findings of the present study showed that PDAC patients exhibited few infiltrating NK cells. Although a high concentration of CD56^+^ NK cells is associated with a good prognosis in PDAC patients who have not received adjuvant therapy before surgery, it is not an independent prognostic factor. This can be attributed to the gradual impairment of NK cell function observed during the progression of pancreatic cancer and the inability of circulating NK cells to survive or proliferate after reaching the hypoxic tumor microenvironment (pO_2_ ≤ 1.5%) (7). The current study confirmed the significance of CD56^+^ NK cell infiltration in the prognosis of PDAC.

The role of neutrophils in the microenvironment of pancreatic cancer is also controversial. Wang et al. (34) used tissue chip technology to explore the presence of CD15^+^ neutrophils and CD20^+^ B cells in tumors and reported that they are predictors of shortened postoperative survival. Miksch et al. (35) used conventional SSH technology and reported that CD66b^+^ neutrophil infiltration in PDAC was not significantly associated with patient prognosis, whereas upregulation of CD20^+^ B cells was significantly correlated with an improved prognosis in PDAC patients. Moreover, Takakura et al. (36) used conventional SSH technology and reported that low-density CD66b^+^ neutrophils and high-density CD20^+^ B cells were predictors of a good prognosis in PDAC patients. The differences in these studies can be attributed to bias from tissue sampling using traditional SSH or tissue chip methods. Traditional SSH or tissue chip techniques seem effective and space-saving; however, these methods do not provide a complete picture of the tumor. In the present study, both neutrophils with low CD15^+^ expression and B cells with high CD20^+^ expression were significantly correlated with a good prognosis in PDAC patients. This may be because neutrophils recruit immune cells that promote tumor progression, whereas B cells directly recognize tumor antigens and produce tumor-related antibodies that kill tumor cells by activating the complement system or promoting ADCC effects (9, 11). These findings show that B cells and neutrophils are effective indicators of the prognosis of pancreatic cancer.

The findings of the current study showed that CDX2 is an excellent prognostic factor in PDAC. Previous studies have reported inconsistent results regarding the patterns of CDX2 expression in PDAC. Werling et al., Chu et al., and Kaimaktchiev et al. reported that CDX2 is heterogeneously expressed in PDAC, with rates of 32 (7/22), 22 (10/46), and 15% (3/20), respectively (37–39). However, these findings were contrary to those from other reports that showed no CDX2 expression in PDAC (40). The findings from the current study are consistent with the findings by Xiao et al. (24), i.e., CDX2 expression is a good predictor in PDAC patients. This is possibly because CDX2 regulates the tumor suppressor gene miR-615-5p and inhibits pancreatic cancer cell proliferation (41). In addition, the findings of this study showed that DPC4 negativity was an independent prognostic predictor for shortened OS in PDAC patients. These findings were consistent with the findings of Biankin et al. (25) and Blackford et al. (42) and can be attributed to the fact that DPC4 is a specific PDAC suppressor gene whose deletion induces upregulation of PGK1 in PDAC, thus enhancing glycolysis and tumor aggressiveness (43).

Nomograms have been used to explore the prognosis of patients across various clinical oncology settings (44). Nomograms integrate multiple prognostic determinants, including genes, molecules, and clinicopathological parameters, and can use relatively simple output forms to calculate and visualize the numerical probability of clinical events. Therefore, they are more effective in prognosis prediction than traditional TNM staging system (45). Although nomograms have been widely used to predict the prognosis of pancreatic cancer patients (46–49), several factors limit their efficiency. Currently, no nomogram has been constructed for PDAC patients based on immune cell parameters and clinicopathological parameters in LSH. Notably, the traditional TNM staging system does not fully consider the host’s immune response in the TME during PDAC progression and does not provide adequate biological information regarding the prognosis of PDAC patients. The nomogram constructed and verified in the present study was superior to the traditional TNM staging system in predicting the prognosis of PDAC patients. The nomogram incorporated information on CD4, CD8, CD15, PDL1, and DPC4, which are closely associated with PDAC immunotherapy and driver mutations, and the previously neglected CDX2. The nomogram model developed in the present study based on clinicopathological parameters and immune cell parameters in the TME can be used clinically to select appropriate treatment options for PDAC patients and to evaluate their specific survival rates at 6 months, 1 year and 2 years.

In summary, this is the first study to report a panoramic view of the immune cell composition in the pancreatic cancer TME based on LSH technology. This approach is superior in predicting the prognosis of PDAC patients compared with SSH. The findings of this study show that pTNM stage, CD4LSH, CD8LSH, CD15LSH, PDL1, CDX2, and DPC4 are independent factors for predicting the prognosis of PDAC patients. This study proposes a combination of six variables that guarantees excellent prognostic prediction for PDAC patients. Moreover, a highly accurate nomogram model was constructed and verified to be effective in clinical decision-making. However, the application of LSH in the prediction of PDAC prognosis should be explored further.

## Data Availability Statement

The original contributions presented in the study are included in the article/**Supplementary Material**. Further inquiries can be directed to the corresponding authors.

## Ethics Statement

The studies involving human participants were reviewed and approved by the Ethics Committee of Changhai Hospital Affiliated to the Second Military Medical University. The patients/participants provided their written informed consent to participate in this study.

## Author Contributions

GD, HJ, YL, and JZ conceptualized and designed the study. GD performed experiments, followed the patient’s survival, and wrote and reviewed the first draft of the manuscript. GD, MG, YY, CS, SW, XL, and JW acquired, analyzed, and interpreted the data and performed statistical analyses. All authors contributed to the article and approved the submitted version.

## Funding

This work was supported by funding from the National Natural Science Foundation of China (Nos. 81972282, 81972683).

## Conflict of Interest

The authors declare that the research was conducted in the absence of any commercial or financial relationships that could be construed as potential conflict of interest.

## Supplementary Material

Supplementary Figure 1 Panoramic scanning images of CD4, CD8, CD15, CD20 and CD56 in LSH of PDAC after immunohistochemical staining. LSH, large-section histopathology; PDAC, pancreatic ductal adenocarcinoma.

Supplementary Figure 2 Panoramic scanning images of CD4, CD8, CD15, CD20 and CD56 in SSH of PDAC after immunohistochemical staining. SSH, small-section histopathology; PDAC, pancreatic ductal adenocarcinoma.

Supplementary Figure 3 H&E images for LSH and SSH and H&E panoramic scan of LSH in PDAC. LSH, large-section histopathology; SSH, small-section histopathology; PDAC, pancreatic ductal adenocarcinoma.

Supplementary Figure 4 Kaplan-Meier survival analysis curves according to 14 **(A–N)** variables that were associated with PDAC prognosis. PDAC, pancreatic ductal adenocarcinoma.
