# Supplementary figures and images for: Large-Section Histopathology Can Better Indicate the Immune Microenvironment and Predict the Prognosis of Pancreatic Ductal Adenocarcinoma Than Small-Section Histopathology
Source: Front Oncol. 2021 Jul 12;11:694933. doi: 10.3389/fonc.2021.694933 (PMC8340684; doi:10.3389/fonc.2021.694933)

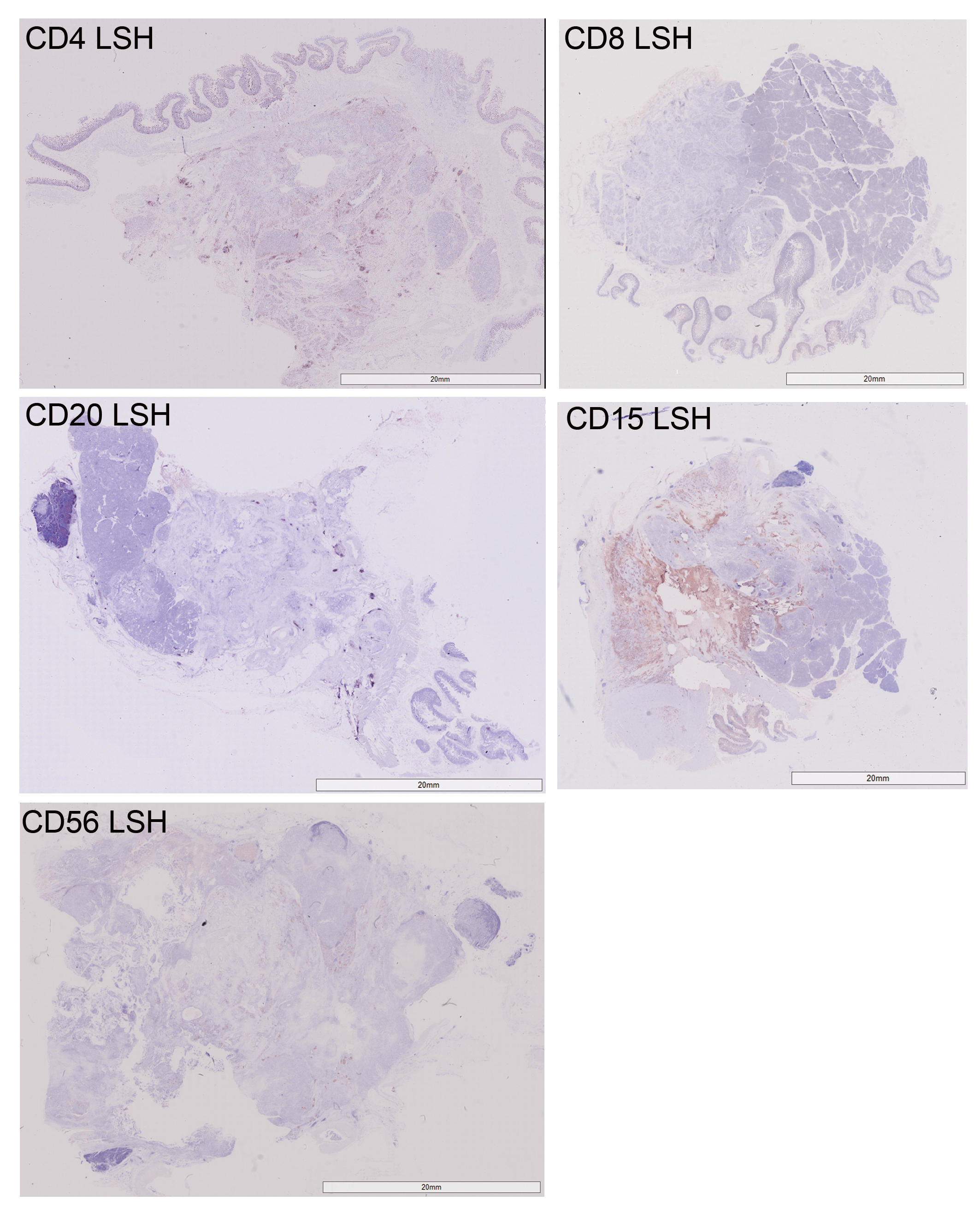

Supplement: Supplementary Figure 1 — Panoramic scanning images of CD4, CD8, CD15, CD20 and CD56 in LSH of PDAC after immunohistochemical staining. LSH, large-section histopathology; PDAC, pancreatic ductal adenocarcinoma. [file Image_1.tif]

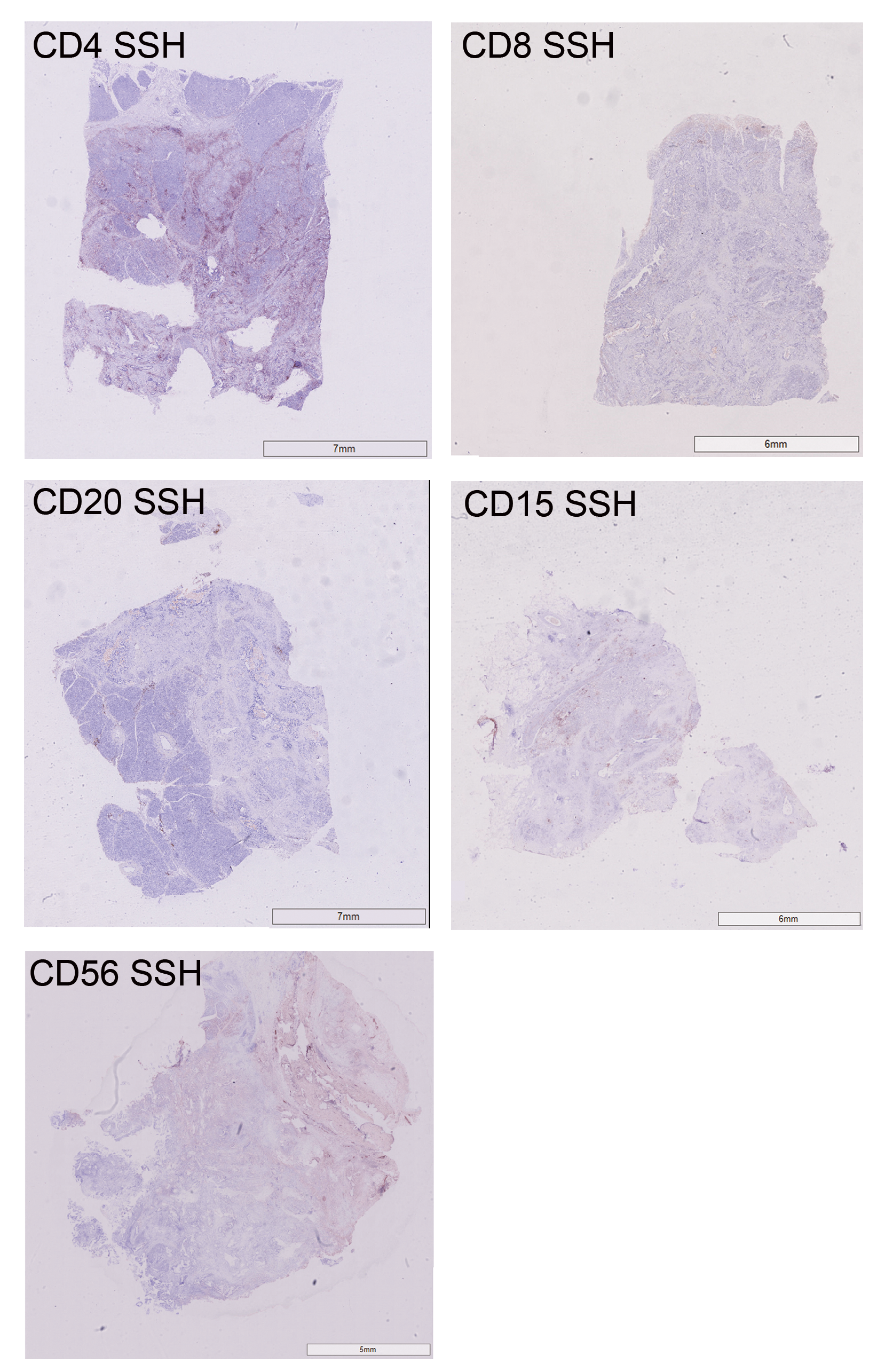

Supplement: Supplementary Figure 2 — Panoramic scanning images of CD4, CD8, CD15, CD20 and CD56 in SSH of PDAC after immunohistochemical staining. SSH, small-section histopathology; PDAC, pancreatic ductal adenocarcinoma. [file Image_2.tif]

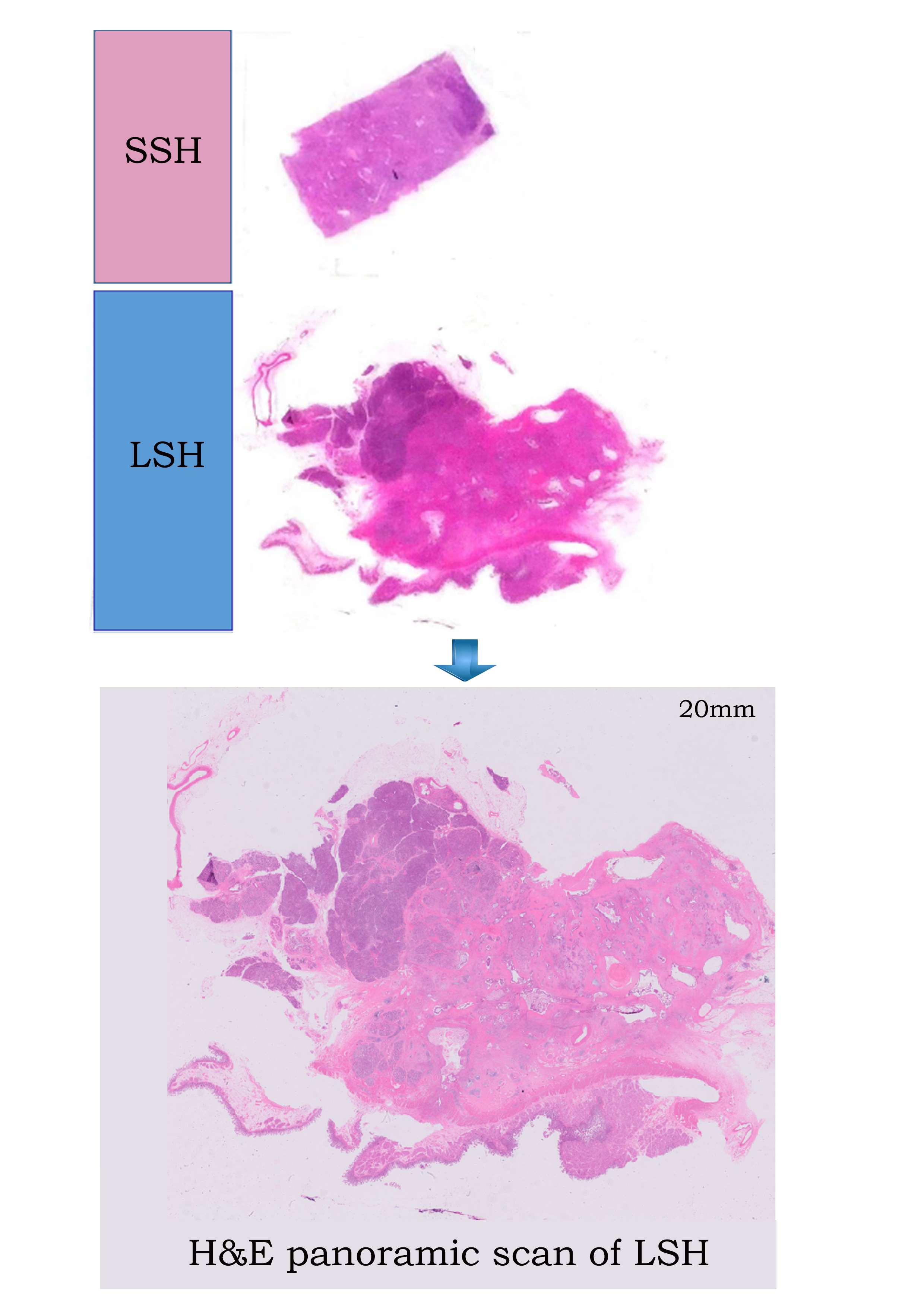

Supplement: Supplementary Figure 3 — H&E images for LSH and SSH and H&E panoramic scan of LSH in PDAC. LSH, large-section histopathology; SSH, small-section histopathology; PDAC, pancreatic ductal adenocarcinoma. [file Image_3.jpeg]

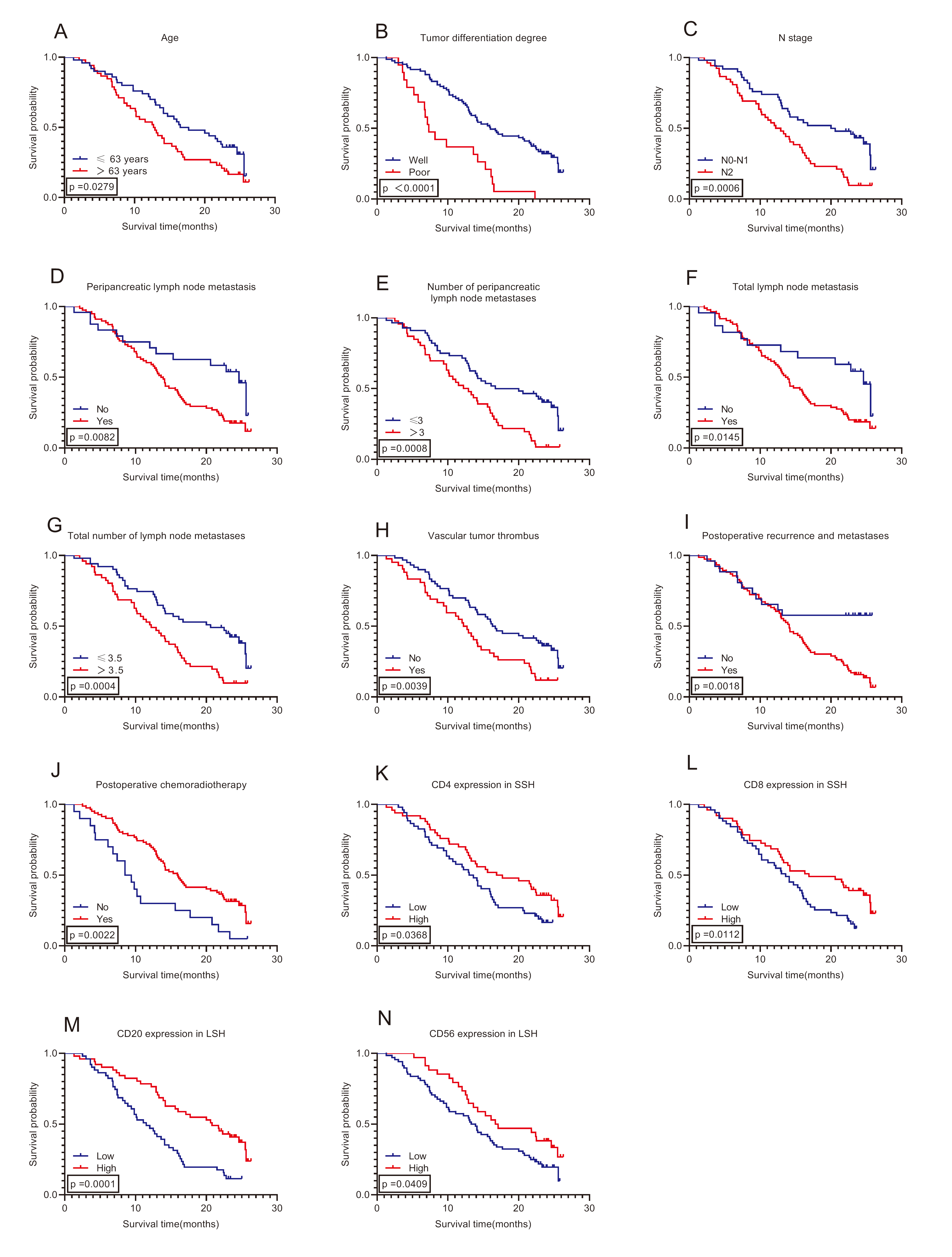

Supplement: Supplementary Figure 4 — Kaplan-Meier survival analysis curves according to 14 (A–N) variables that were associated with PDAC prognosis. PDAC, pancreatic ductal adenocarcinoma. [file Image_4.tif]
